# 2-Amino-3-nitro­pyridinium hydrogen oxalate

**DOI:** 10.1107/S1600536809008666

**Published:** 2009-03-19

**Authors:** Samah Akriche, Mohamed Rzaigui

**Affiliations:** a Laboratoire de Chimie des Matériaux, Faculté des Sciences de Bizerte, 7021 Zarzouna Bizerte, Tunisia

## Abstract

In the non-centrosymetric title compound, C_5_H_6_N_3_O_2_^+^·C_2_HO_4_^−^, the hydrogen oxalate anions form corrugated chains parallel to the *c* axis, linked by O—H⋯O hydrogen bonds. The 2-amino-3-nitro­pyridinium cations are anchored between theses chains by N—H⋯O and C—H⋯O hydrogen bonds and van der Waals and electrostatic inter­actions, creating a three-dimensional network.

## Related literature

For related structures, see: Akriche & Rzaigui (2000 ▶, 2009 ▶); Le Fur *et al.* (1998 ▶); Nicoud *et al.* (1997 ▶); For a discussion of hydrogen bonding, see: Desiraju (1989 ▶, 1995 ▶). 
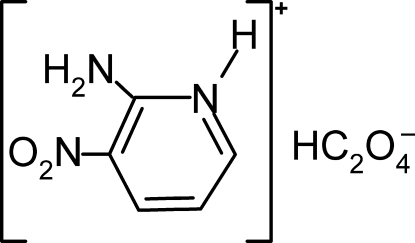


## Experimental

### 

#### Crystal data

C_5_H_6_N_3_O_2_^+^·C_2_HO_4_^−^
*M* *_r_* = 229.16
Orthorhombic, 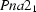

*a* = 15.268 (4) Å
*b* = 6.921 (3) Å
*c* = 8.807 (2) Å
*V* = 930.6 (5) Å^3^
*Z* = 4
Mo *K*α radiation
μ = 0.15 mm^−1^
*T* = 293 K
0.33 × 0.25 × 0.21 mm

#### Data collection

Enraf–Nonius Turbo CAD-4 diffractometer
Absorption correction: none
2228 measured reflections
1190 independent reflections
1003 reflections with *I* > 2σ(*I*)
*R*_int_ = 0.020
2 standard reflections frequency: 120 min intensity decay: 1%

#### Refinement

*R*[*F* ^2^ > 2σ(*F* ^2^)] = 0.034
*wR*(*F* ^2^) = 0.088
*S* = 1.06
1190 reflections
147 parameters
1 restraint
H-atom parameters constrained
Δρ_max_ = 0.33 e Å^−3^
Δρ_min_ = −0.20 e Å^−3^

### 

Data collection: *CAD-4 EXPRESS* (Enraf–Nonius, 1994 ▶); cell refinement: *CAD-4 EXPRESS*; data reduction: *XCAD4* (Harms & Wocadlo, 1996 ▶); program(s) used to solve structure: *SHELXS97* (Sheldrick, 2008 ▶); program(s) used to refine structure: *SHELXL97* (Sheldrick, 2008 ▶); molecular graphics: *ORTEP-3* (Farrugia, 1997 ▶) and *DIAMOND* (Brandenburg & Putz, 2005 ▶); software used to prepare material for publication: *WinGX* (Farrugia, 1999 ▶).

## Supplementary Material

Crystal structure: contains datablocks I, global. DOI: 10.1107/S1600536809008666/kj2116sup1.cif

Structure factors: contains datablocks I. DOI: 10.1107/S1600536809008666/kj2116Isup2.hkl

Additional supplementary materials:  crystallographic information; 3D view; checkCIF report

## Figures and Tables

**Table 1:** Hydrogen-bond geometry (Å, °)

*D*—H⋯*A*	*D*—H	H⋯*A*	*D*⋯*A*	*D*—H⋯*A*
O3—H3⋯O2^i^	0.82	1.82	2.632 (2)	171
N1—H1⋯O2	0.86	1.85	2.706 (3)	175
N2—H2*A*⋯O1	0.86	1.99	2.837 (3)	170
N2—H2*B*⋯O5	0.86	2.09	2.673 (3)	124
N2—H2*B*⋯O4^ii^	0.86	2.51	3.188 (3)	136
C3—H3*A*⋯O5^iii^	0.93	2.44	3.178 (3)	136
C4—H4⋯O1^iv^	0.93	2.33	3.258 (3)	174
C5—H5⋯O6^v^	0.93	2.57	3.262 (3)	132

Symmetry codes: (i) 
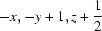
; (ii) 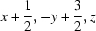; (iii) 
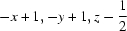
; (iv) 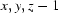; (v) 
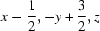
.

**Fig. 1.**
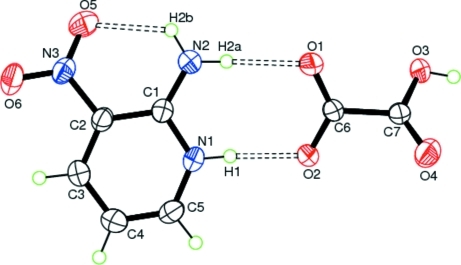
View of the title compound with the atom-labelling scheme. Displacement ellipsoids are drawn at the 50% probability level. Hydrogen bonds are represented as dashed lines.

**Fig. 2.**
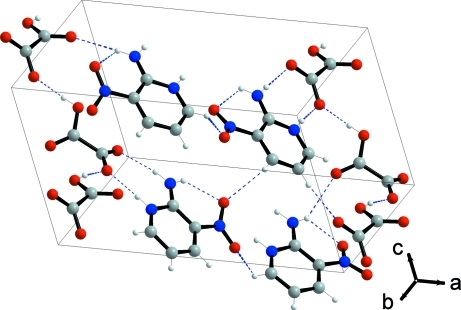
A perspective view of the packing of the title compound. Hydrogen bonds are represented as dashed lines.

## supplementary crystallographic information

### Comment

The search for new molecular materials for the non-linear optics lies at the basis of our ongoing study of 2-amino-3-nitropyridinium salts. Our strategy is aimed at the production of very cohesive non-centrosymmetric packing of chromophores. We have previously reported two centrosymetric structures of 2-amino-3-nitropyridinium (Akriche & Rzaigui, 2000; Akriche & Rzaigui, 2009). We report here a new non-centrosymmetric structure, 2-amino-3-nitropyridinium hydrogenoxalate.

The asymmetric unit of the title compound consists of one (HC_2_O_4_)^-^ anion and one (2-NH_2_-3-NO_2_C_5_H_3_NH)^+^ cation (Fig. 1). In the hydrogenoxalate (HC_2_O_4_)^-^, the H atom is located at O3 as is also indicated by elongation of the corresponding C—O distance [O3—C7 is 1.314 (3) Å]. The bond length of C6—C7 is relatively long [1.545 (3) Å] as expected for an oxalate anion. In the 2-amino-3-nitropyridinium cation, nitro and amino groups are *ortho* to one another, which explains the presence of the intra-cation contact N2—H2B···O5 (Le Fur *et al.*, 1998; Nicoud *et al.*,1997).

The structure projection in Fig. 2 shows that the oxalate ions are organized in corrugated chains extending along the *c* axis. The cations are located between these chains and manifest multiple H-bonds. In fact, in this structure there are three categories of H-bond (Table 1), O—H···O, N—H···O and C—H···O. Within each oxalate chain, the (HC_2_O_4_)^-^ groups are interconnected by strong O—H···O hydrogen bonds. These chains are themselves interconnected by N—H···O interactions originating from the NH^+^ and NH_2_ groups of the cations. It is worth noticing the presence of long C—H···O contacts (Desiraju, 1989; Desiraju, 1995) occurring between cations and between cations and anions. The density of this H-bond scheme constitutes probably the main factor responsible for the formation of a non-centrosymetric material.

### Experimental

An aqueous solution containing 0.004 mol of H_2_C_2_O_4_ in 10 ml of water, was added to 0.004 mol of 2-amino-3-nitropyridine in 20 ml of pure acetic acid. The obtained yellow solution was stirred at 333 K for 10 min and then left to stand at room temperature. Yellow single crystals of the title compound were obtained after some days.

### Crystal data

**Table:**

C_5_H_6_N_3_O_2_^+^·C_2_HO_4_^−^	*D*_x_ = 1.636 Mg m^−^^3^
*M**_r_* = 229.16	Mo *K*α radiation, λ = 0.71073 Å
Orthorhombic, *P**n**a*2_1_	Cell parameters from 25 reflections
*a* = 15.268 (4) Å	θ = 9–11°
*b* = 6.921 (3) Å	µ = 0.15 mm^−^^1^
*c* = 8.807 (2) Å	*T* = 293 K
*V* = 930.6 (5) Å^3^	Rectangular prism, yellow
*Z* = 4	0.33 × 0.25 × 0.21 mm
*F*(000) = 472	

### Data collection

**Table:**

Enraf–Nonius Turbo CAD-4 diffractometer	*R*_int_ = 0.020
Radiation source: Enraf–Nonius FR590	θ_max_ = 28.0°, θ_min_ = 2.7°
graphite	*h* = −20→0
Nonprofiled ω scans	*k* = −7→9
2228 measured reflections	*l* = −11→0
1190 independent reflections	2 standard reflections every 120 min
1003 reflections with *I* > 2σ(*I*)	intensity decay: 1%

### Refinement

**Table:**

Refinement on *F*^2^	Secondary atom site location: difference Fourier map
Least-squares matrix: full	Hydrogen site location: inferred from neighbouring sites
*R*[*F*^2^ > 2σ(*F*^2^)] = 0.034	H-atom parameters constrained
*wR*(*F*^2^) = 0.088	*w* = 1/[σ^2^(*F*_o_^2^) + (0.0559*P*)^2^ + 0.0295*P*] where *P* = (*F*_o_^2^ + 2*F*_c_^2^)/3
*S* = 1.06	(Δ/σ)_max_ = 0.001
1190 reflections	Δρ_max_ = 0.33 e Å^−^^3^
147 parameters	Δρ_min_ = −0.20 e Å^−^^3^
1 restraint	Extinction correction: *SHELXL97* (Sheldrick, 2008), Fc^*^=kFc[1+0.001xFc^2^λ^3^/sin(2θ)]^-1/4^
Primary atom site location: structure-invariant direct methods	Extinction coefficient: 0.053 (6)

### Special details

**Table:**

Geometry. H atoms were treated as riding, with C—H = 0.93 A °, N—H = 0.86 A ° and O—H = 0.82 A °, and with *U*_iso_(H) = 1.2Ueq(C,*N*) and 1.5Ueq(O). All e.s.d.'s (except the e.s.d. in the dihedral angle between two l.s. planes) are estimated using the full covariance matrix. The cell e.s.d.'s are taken into account individually in the estimation of e.s.d.'s in distances, angles and torsion angles; correlations between e.s.d.'s in cell parameters are only used when they are defined by crystal symmetry. An approximate (isotropic) treatment of cell e.s.d.'s is used for estimating e.s.d.'s involving l.s. planes.
Refinement. Refinement of *F*^2^ against ALL reflections. The weighted *R*-factor *wR* and goodness of fit *S* are based on *F*^2^, conventional *R*-factors *R* are based on *F*, with *F* set to zero for negative *F*^2^. The threshold expression of *F*^2^ > σ(*F*^2^) is used only for calculating *R*-factors(gt) *etc*. and is not relevant to the choice of reflections for refinement. *R*-factors based on *F*^2^ are statistically about twice as large as those based on *F*, and *R*- factors based on ALL data will be even larger.

### Fractional atomic coordinates and isotropic or equivalent isotropic displacement parameters (Å^2^)

**Table:**

	*x*	*y*	*z*	*U*_iso_*/*U*_eq_	
O1	0.18864 (12)	0.6389 (3)	0.4311 (2)	0.0512 (5)	
O2	0.10055 (11)	0.5552 (3)	0.2423 (2)	0.0408 (4)	
O3	0.05130 (11)	0.5172 (3)	0.6129 (2)	0.0391 (4)	
H3	0.0051	0.5043	0.6593	0.059*	
O4	−0.03139 (11)	0.6833 (3)	0.4481 (2)	0.0494 (5)	
O5	0.49613 (12)	0.5813 (3)	0.1226 (3)	0.0532 (5)	
O6	0.52023 (11)	0.6750 (3)	−0.1071 (3)	0.0545 (5)	
N1	0.23161 (12)	0.5903 (3)	0.0374 (3)	0.0375 (5)	
H1	0.1920	0.5818	0.1065	0.045*	
N2	0.33259 (15)	0.6060 (3)	0.2291 (3)	0.0462 (6)	
H2A	0.2900	0.6012	0.2930	0.055*	
H2B	0.3857	0.6133	0.2612	0.055*	
N3	0.47122 (12)	0.6244 (3)	−0.0050 (3)	0.0367 (5)	
C1	0.31649 (14)	0.6020 (3)	0.0827 (3)	0.0321 (5)	
C2	0.37793 (14)	0.6148 (3)	−0.0380 (3)	0.0318 (5)	
C3	0.35201 (16)	0.6188 (4)	−0.1871 (3)	0.0363 (5)	
H3A	0.3936	0.6300	−0.2638	0.044*	
C4	0.26384 (17)	0.6061 (4)	−0.2236 (3)	0.0437 (6)	
H4	0.2452	0.6078	−0.3241	0.052*	
C5	0.20572 (16)	0.5911 (4)	−0.1078 (3)	0.0421 (6)	
H5	0.1463	0.5811	−0.1298	0.050*	
C6	0.11677 (14)	0.5994 (3)	0.3773 (3)	0.0303 (5)	
C7	0.03636 (14)	0.6065 (3)	0.4838 (3)	0.0302 (5)	

### Atomic displacement parameters (Å^2^)

**Table:**

	*U*^11^	*U*^22^	*U*^33^	*U*^12^	*U*^13^	*U*^23^
O1	0.0315 (8)	0.0930 (14)	0.0292 (9)	−0.0146 (10)	−0.0004 (7)	−0.0040 (10)
O2	0.0286 (7)	0.0684 (11)	0.0254 (8)	−0.0040 (8)	0.0007 (7)	−0.0037 (9)
O3	0.0300 (8)	0.0618 (12)	0.0256 (7)	0.0005 (8)	0.0063 (7)	0.0065 (8)
O4	0.0346 (9)	0.0660 (12)	0.0475 (11)	0.0110 (8)	0.0040 (8)	0.0115 (10)
O5	0.0342 (9)	0.0727 (13)	0.0528 (12)	0.0094 (9)	−0.0082 (9)	0.0076 (11)
O6	0.0330 (9)	0.0779 (13)	0.0526 (12)	−0.0118 (9)	0.0125 (8)	−0.0050 (11)
N1	0.0243 (9)	0.0522 (14)	0.0361 (12)	−0.0015 (8)	0.0039 (8)	−0.0004 (9)
N2	0.0317 (10)	0.0773 (18)	0.0296 (11)	−0.0022 (10)	0.0002 (8)	0.0046 (11)
N3	0.0259 (9)	0.0401 (10)	0.0440 (12)	0.0007 (8)	0.0035 (9)	−0.0039 (9)
C1	0.0271 (10)	0.0372 (12)	0.0319 (12)	−0.0008 (9)	0.0024 (9)	0.0020 (9)
C2	0.0252 (10)	0.0356 (11)	0.0347 (12)	0.0013 (8)	0.0024 (9)	−0.0014 (10)
C3	0.0352 (12)	0.0427 (14)	0.0311 (12)	0.0002 (10)	0.0057 (10)	−0.0004 (10)
C4	0.0407 (13)	0.0592 (17)	0.0311 (13)	0.0026 (11)	−0.0038 (10)	−0.0018 (11)
C5	0.0271 (10)	0.0585 (16)	0.0406 (15)	0.0020 (10)	−0.0049 (10)	−0.0044 (12)
C6	0.0279 (10)	0.0391 (11)	0.0240 (10)	−0.0029 (9)	0.0015 (9)	0.0033 (9)
C7	0.0263 (10)	0.0382 (11)	0.0261 (11)	−0.0040 (8)	−0.0003 (8)	−0.0026 (9)

### Geometric parameters (Å, °)

**Table:**

O1—C6	1.226 (3)	N2—H2A	0.8600
O2—C6	1.252 (3)	N2—H2B	0.8600
O3—C7	1.314 (3)	N3—C2	1.455 (3)
O3—H3	0.8200	C1—C2	1.420 (3)
O4—C7	1.205 (3)	C2—C3	1.372 (3)
O5—N3	1.223 (3)	C3—C4	1.387 (3)
O6—N3	1.221 (3)	C3—H3A	0.9300
N1—C5	1.338 (4)	C4—C5	1.356 (4)
N1—C1	1.358 (3)	C4—H4	0.9300
N1—H1	0.8600	C5—H5	0.9300
N2—C1	1.313 (3)	C6—C7	1.545 (3)
			
C7—O3—H3	109.5	C2—C3—C4	120.0 (2)
C5—N1—C1	124.2 (2)	C2—C3—H3A	120.0
C5—N1—H1	117.9	C4—C3—H3A	120.0
C1—N1—H1	117.9	C5—C4—C3	117.8 (2)
C1—N2—H2A	120.0	C5—C4—H4	121.1
C1—N2—H2B	120.0	C3—C4—H4	121.1
H2A—N2—H2B	120.0	N1—C5—C4	121.7 (2)
O6—N3—O5	123.8 (2)	N1—C5—H5	119.1
O6—N3—C2	117.8 (2)	C4—C5—H5	119.1
O5—N3—C2	118.5 (2)	O1—C6—O2	126.8 (2)
N2—C1—N1	117.9 (2)	O1—C6—C7	118.0 (2)
N2—C1—C2	127.6 (2)	O2—C6—C7	115.25 (19)
N1—C1—C2	114.5 (2)	O4—C7—O3	125.6 (2)
C3—C2—C1	121.8 (2)	O4—C7—C6	122.5 (2)
C3—C2—N3	118.2 (2)	O3—C7—C6	111.85 (19)
C1—C2—N3	120.0 (2)		
			
C5—N1—C1—N2	177.8 (3)	C1—C2—C3—C4	−1.3 (4)
C5—N1—C1—C2	−0.2 (4)	N3—C2—C3—C4	178.7 (2)
N2—C1—C2—C3	−176.6 (3)	C2—C3—C4—C5	0.4 (4)
N1—C1—C2—C3	1.2 (3)	C1—N1—C5—C4	−0.7 (5)
N2—C1—C2—N3	3.3 (4)	C3—C4—C5—N1	0.6 (4)
N1—C1—C2—N3	−178.8 (2)	O1—C6—C7—O4	134.2 (3)
O6—N3—C2—C3	14.8 (3)	O2—C6—C7—O4	−45.0 (3)
O5—N3—C2—C3	−165.1 (2)	O1—C6—C7—O3	−46.9 (3)
O6—N3—C2—C1	−165.2 (2)	O2—C6—C7—O3	133.9 (2)
O5—N3—C2—C1	15.0 (3)		

### Hydrogen-bond geometry (Å, °)

**Table:**

*D*—H···*A*	*D*—H	H···*A*	*D*···*A*	*D*—H···*A*
O3—H3···O2^i^	0.82	1.82	2.632 (2)	171
N1—H1···O2	0.86	1.85	2.706 (3)	175
N2—H2A···O1	0.86	1.99	2.837 (3)	170
N2—H2B···O5	0.86	2.09	2.673 (3)	124
N2—H2B···O4^ii^	0.86	2.51	3.188 (3)	136
C3—H3A···O5^iii^	0.93	2.44	3.178 (3)	136
C4—H4···O1^iv^	0.93	2.33	3.258 (3)	174
C5—H5···O6^v^	0.93	2.57	3.262 (3)	132

Symmetry codes: (i) −*x*, −*y*+1, *z*+1/2; (ii) *x*+1/2, −*y*+3/2, *z*; (iii) −*x*+1, −*y*+1, *z*−1/2; (iv) *x*, *y*, *z*−1; (v) *x*−1/2, −*y*+3/2, *z*.
